# Development of a Rheology Die and Flow Characterization of Gas-Containing Polymer Melts

**DOI:** 10.3390/polym13193305

**Published:** 2021-09-27

**Authors:** Clemens Kastner, Dominik Altmann, Eva Kobler, Georg Steinbichler

**Affiliations:** 1 Institute of Polymer Injection Molding and Process Automation, Johannes Kepler University Linz, Altenberger Strasse 69, 4040 Linz, Austria; dominik.altmann@jku.at (D.A.); eva.kobler@jku.at (E.K.); georg.steinbichler@jku.at (G.S.); 2 Competence Center CHASE GmbH, Altenberger Strasse 69, 4040 Linz, Austria; 3 Kompetenzzentrum Holz GmbH (Wood K Plus)—Biobased Composites and Processes, Altenberger Strasse 69, 4040 Linz, Austria

**Keywords:** polymer processing, foam injection molding, polypropylene, viscosity, ultrasound, blowing agents, sustainability

## Abstract

We present a novel measurement die for characterizing the flow behavior of gas-containing polymer melts. The die is mounted directly on the injection-molding cylinder in place of the mold cavity and thus enables near-process measurement of viscosity (i.e., under the conditions that would be present were a mold attached). This integration also resolves the issue of keeping gas-containing polymer melts under pressure during measurement to prevent desorption. After thermal characterization of the die, various correction approaches were compared against each other to identify the most suitable one for our case. We conducted measurements using polypropylene in combination with two different chemical blowing agents. Increasing the blowing-agent content to up to 6% revealed an interestingly low influence of gases on melt viscosity, which was confirmed by elongational viscosity measurements. For verification, we compared our results to corresponding measurements taken on a high-pressure capillary rheometer and found that they were in excellent agreement. Our die cannot only be used for rheological characterization. Combined with ultrasound sensors, it provides an innovative way of measuring the volumetric flow rate. This development represents an important step in improving the sustainability of gas-containing polymer processing.

## 1. Introduction

Against the backdrop of climate change, sustainability has become a prime objective [1,2]. The importance of polymeric materials is growing rapidly. In 2015, with a total worldwide production of 322 million metric tons, the two most important polyolefins (polyethylene (PE) and polypropylene (PP)) had a combined market share of over 50% [3]. With an eye on sustainability, this can be explained by (i) their low density and light weight, (ii) the possibility of integrating many functions into a single component, and (iii) their energy-saving and thus economical production and processing.

Aspect (iii) in particular is closely related to the flowability (and thus the viscosity) of polymer melts. Understanding the rheology of gas-containing polymer melts is therefore essential in polymer processing (for machine selection, mold design, etc.). Further, since accurate data also improve the capabilities of simulations (e.g., in injection molding), the general objective is to measure viscosity as close to the process as possible.

Gas-containing polymer melts must be kept under sufficient pressure to prevent the gases from desorbing. Broadly, according to Henry’s law, the pressures necessary to maintain solutions of gases such as carbon dioxide (CO_2_) and nitrogen (N_2_) in PP are of the order of 100 bar [4,5]. Application of such pressures in combination with precise control of gas contents is challenging. State-of-the-art high-pressure capillary rheometers (HPCRs) can maintain the necessary pressures prior to measurement (i.e., during heating and melting), but lack proper sealing and do not usually allow mixing. Methods using rotational rheometers allow gas pressures to be applied, but the shear rates are insufficient for injection molding processes, and gas content can only be controlled via saturation pressures (i.e., no direct control possible).

Park and Dealy [6] used a high-pressure sliding-plate rheometer to investigate the rheology of high-density polyethylene (HDPE) in combination with supercritical CO_2_ and N_2_ at low shear rates of up to 100 s^−1^. They measured a strong plasticizing effect of CO_2_ and a much weaker one of N_2_. However, they concluded that at equal molar concentrations both gases have the same viscosity-reducing effect and that the differences are due to the poorer solubility of N_2_. A rotational rheometer in a pressure cell was also used by Raps et al. [7]. Investigating a solution of CO_2_ in high-melt-strength PP (HMS-PP), they found a more than 50% reduction in viscosity at a gas concentration of 17%.

Lee et al. [8] studied the effect of supercritical CO_2_ on polyethylene/polystyrene blends in a twin-screw extruder at 195 °C. A gas concentration of 5% caused a reduction in viscosity by approximately 40%. In addition, they observed reduced shear-thinning at increasing gas contents. In a subsequent study [9], they investigated the influence of supercritical CO_2_ on polystyrene (PS) at 220 °C, using a single-screw extruder. Their results indicate a 70–75% viscosity reduction at a gas concentration of 4%. In a third study [10], they also showed considerable differences in the influence of supercritical CO_2_ on various polymers (again in an extrusion process). According to their results, PS is much more sensitive to gas content than PE. As can be seen, all these studies used extrusion equipment. Influences of process characteristics typical in injection molding (discontinuity, standstill times, a reciprocating screw, etc.) cannot be investigated.

The literature on use of injection molding machines in this context is limited. Mei et al. [11] investigated and evaluated the gas loading capability of polymer melts (PP and HDPE blends) under atmospheric conditions using a modified torque rheometer in injection molding processing. In addition, an attempt was made to illustrate the effects by morphological studies using SEM (scanning electron microscopy). Neat polymers (PP and PS) were investigated by Aho and Syrjälä [12]. They designed a slit die that can be attached directly to a plasticizing unit, and obtained satisfactory (near-process) results. In terms of process proximity, Qin et al. [13] developed a sophisticated approach. They instrumented the mold cavity (slot geometry) of an injection molding machine with pressure transducers and studied the effect of endothermic and exothermic chemical blowing agents (CBAs) on low-density PE (LDPE) and thermoplastic polyolefins (TPOs). For example, a 31% decrease in viscosity was measured at 5% exothermic CBA for TPO at 250 °C. In general, exothermic CBAs led to lower viscosities compared to endothermic ones.

Interestingly, although N_2_ is frequently used in physical foam injection molding processes, very few publications on its influence on the viscosity of polymer melts are available. Only Qin et al. [13] conducted trials with N_2_ as a physical blowing agent. At gas concentrations that are common in industrial applications (here 0.4%), a negligible reduction in viscosity was measured. Excessive gas contents of up to 2.3% were required for a noticeable improvement in flow behavior, but the effect was still below that achieved with CO_2_.

The lack of near-process measurement data motivated us to develop a rheology die for characterizing gas-containing polymer melts in injection molding machines. Our die is mounted directly on the plasticizing cylinder and replaces the mold cavity. This proximity to the material to be measured allows: (i) capturing of processes as they occur under real conditions inside the cylinder, and (ii) accurate description of the flows present during mold filling. The data thus collected provide a close representation of reality and also play a crucial part in accurate simulations.

## 2. Concept and Design of a Novel Shear-Viscosity Die

**Figure 1:**
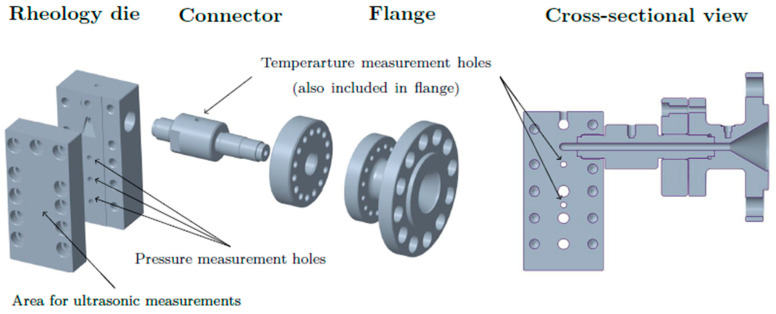
CAD model of our slit die for injection molding: exploded (**left**) and cross-sectional (**right**) views.

Figure 1 shows a representation of the computer-aided design (CAD) model of the setup we developed, including the die and components necessary for mounting. Flange and connector facilitate proper vertical alignment of the die. Calculation of the viscosity requires the pressure gradient and the flow rate of the polymer melt. The former can be obtained based on screw speed, but should also be monitored via ultrasound sensors, which are attached to the plane surface indicated in Figure 1. The latter is measured via three pressure transducers along the slit. The boreholes for the pressure transducers are designed to allow application of pressure transducers from both Kistler GmbH (Vienna, Austria) and Gefran S.p.A. (Provaglio d’Iseo, Italy).

In its current configuration, the rheology die has a flow channel with a rectangular cross section. The dimensions of the slit are summarized in Table 1. To prevent influences of the edges on the flow field, the slit must be sufficiently wide (width ≥ 10*height [12]). Hereafter, P1, P2 and P3 denote the pressure transducers in the flow direction (i.e., from top to bottom). It is well known that the compressibility of polymer melts increases with increasing gas concentrations [14]. This might lead to errors when the flow speed in the slit is derived directly from screw position or velocity. To eliminate these potential inaccuracies, ultrasound sensors should be used. For measurement of the pressure profile along the slit, a valve that generates back-pressure is to be integrated at the exit of the die.

## 3. Experimental

### 3.1. Shear Viscosity—Apparent Properties

Calculating the viscosities requires geometry of the flow channel, pressure gradient along the channel and flow rate to be known. The flow rate through a die (mounted on an injection-molding machine) is traditionally assumed to be equivalent to the theoretical flow rate caused by screw movement (i.e., assuming proper closing behavior of the backflow barrier and no backflow), which can be calculated by

(1) $$\dot{V}=r_{s}{}^{2}\pi v_{s}\;$$

where $r_{s}$ is the screw radius and $v_{s}$ the axial screw speed.

Based on the flow rate through the channel, the apparent shear rate, ${\dot{\gamma }}_{ap}$, is then obtained as

(2) $${\dot{\gamma }}_{ap}=\frac{6\dot{V}}{wh^{2}}\;$$

The apparent shear stress, $\tau_{ap}$, depends linearly on the pressure gradient, Δp, and is given by

(3) $$\tau_{ap}=\Delta p\frac{h}{2l}\;$$

where l denotes the flow length (i.e., the distance between pressure sensors). From Equations (2) and (3), the apparent viscosity, $\eta_{ap}$, can be calculated according to:

(4) $$\eta_{ap}=\frac{\tau_{ap}}{{\dot{\gamma }}_{ap}}=\frac{\Delta pwh^{3}}{12l\dot{V}}\;$$

### 3.2. Shear Viscosity—Correction of Measurements

In contrast to HPCRs, where the pressure can only be measured in front of (or, when using a counter-pressure device, after) the die, the rheology die presented here allows pressure measurement directly in the channel. Bagley correction becomes unnecessary, as no inlet and outlet pressure losses occur. Meng et al. [15] performed viscosity measurements of PP using various capillary diameters and showed that no wall slip occurs in a 1 mm capillary at 210–230 °C. In our experiments, we therefore assumed that the no-slip condition is valid, which also rendered a Mooney correction unnecessary.

**Figure 2:**
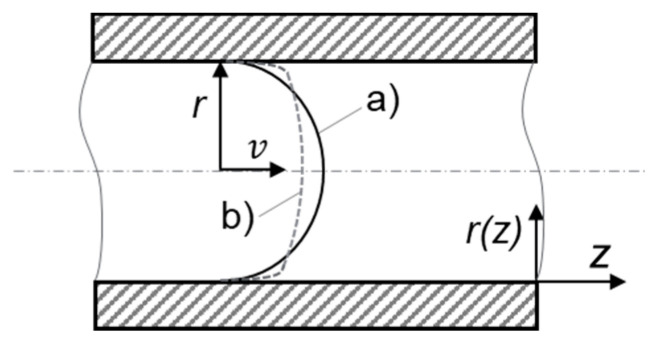
Comparison between parabolic, (a) Newtonian, and non-parabolic, (b) pseudoplastic/non-Newtonian, flow profiles.

Equations (2)–(4) assume Newtonian fluids, but polymers are important representatives of pseudoplastic/shear-thinning (i.e., non-Newtonian) fluids, which means that their viscosities change with shear rate. The velocity profile in the flow channel is therefore not perfectly parabolic, as shown schematically in Figure 2.

Three different approaches to correcting the apparent shear rate are discussed in the following subsections. They are compared against each other to determine the optimal correction approach for our rheology die.

#### 3.2.1. Approach 1—Weissenberg-Rabinowitsch (WR) Correction

The WR correction is the most commonly used approach. The true shear rate, $\dot{\gamma }$, can be obtained by

(5) $$\dot{\gamma }=\frac{2n+1}{3n}{\dot{\gamma }}_{ap}=\left(\frac{2}{3}+\frac{1}{3}\frac{d\log{\dot{\gamma }}_{ap}}{d\log\tau_{ap}}\right){\dot{\gamma }}_{ap}\;$$

where n is the slope of the $\log\tau_{ap}$-$\log{\dot{\gamma }}_{ap}$ curve [16]. In other words, the WR approach corrects for non-Newtonian behavior of the polymer melt by pointwise differentiation of the curve.

As suggested by Müller [17] (p. 58), a discretization scheme according to Equation (6) can be used to make Equation (5) applicable to measured data points. The first and the last (nth) data points can be modeled by Equations (7) and (8), respectively, with v denoting the flow velocity in the channel.

(6) $${\dot{\gamma }}_{i}=\frac{2}{3}{\dot{\gamma }}_{ap_{i}}+\frac{1}{3}\tau_{i}\frac{{\dot{\gamma }}_{ap_{i+1}}-{\dot{\gamma }}_{ap_{i-1}}}{\tau_{ap_{i+1}}-\tau_{ap_{i-1}}}\;$$

(7) $${\dot{\gamma }}_{1}=\frac{2}{3}{\dot{\gamma }}_{ap_{1}}+\frac{1}{3}\tau_{1}\frac{{\dot{\gamma }}_{ap_{2}}}{\tau_{ap_{2}}}\;$$

(8) $${\dot{\gamma }}_{n}={\dot{\gamma }}_{n-1}\frac{v_{n}}{v_{n-1}}\;$$

This discretized and pointwise way of transforming apparent into true shear rates requires a sufficient number of data points. The experiments described in this work therefore involved data acquisition at nine shear rates (orders of magnitude: 10^1^–10^4^ s^−1^).

#### 3.2.2. Approach 2—Schümmer Approximation

**Figure 3:**
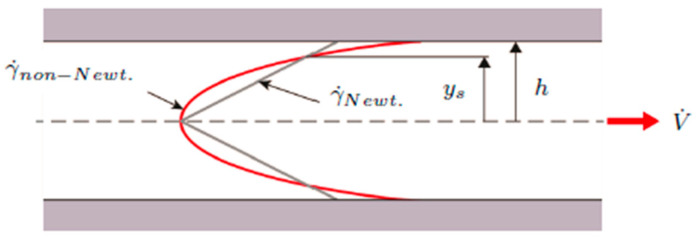
Shear rate profiles for pseudoplastic and Newtonian fluids in a rectangular slit with height h; cited from [19].

This approach, proposed by Schümmer et al. [18], offers a much simpler way of correcting for the non-Newtonian flow profile. It estimates shear rate and viscosity at the distance from the center of the flow profile at which pseudoplastic and Newtonian viscosities coincide. As illustrated in Figure 3, the shear rate in a Newtonian fluid exhibits a linear gradient, while non-Newtonian fluids are described by a curved function.

At a distance $y_{s}$ from the center line, both shear rate functions are equivalent. Aho and Syrjälä [12] showed that this distance can be determined via Equation (9). In their work, they used PP and PS and set the proportionality factor $x^{*}$ to 0.79, which was confirmed by Laun [20] using LDPE. At this point, shear stress and apparent shear rate become $x^{*}\tau$ and $x^{*}{\dot{\gamma }}_{ap}$, respectively.

(9) $$y_{s}=\frac{hx^{*}}{2}\;$$

In contrast to the WR correction, this approach does not correct shear rate and calculate true viscosities. Instead, corrected shear rates are assigned to existing apparent viscosities (see Equation (10)). The resulting combinations of shifted apparent shear rate and apparent viscosities are then true values. While the WR correction affects both shear rate and viscosity, the Schümmer approximation only shifts the viscosity curve horizontally to lower shear rates. Unlike the WR correction, it can be applied to single-point data.

#### 3.2.3. Approach 3—Variant of WR Correction

This correction approach was also used by Kadijk and Van Den Brule [21]. Calculation of the slope, s, in the $\log\eta_{ap}$-$\log{\dot{\gamma }}_{ap}$ curve via Equation (10) allows subsequent derivation of the true shear rate, $\dot{\gamma }$, and the true viscosity, η, according to Equations (11) and (12), respectively.

(10) $$s=\frac{d\log\eta_{ap}}{d\log{\dot{\gamma }}_{ap}}\;$$

(11) $$\eta =\eta_{ap}\frac{2s+3}{3s+3}\;$$

(12) $$\dot{\gamma }={\dot{\gamma }}_{ap}\frac{3s+3}{2s+3}\;$$

### 3.3. Elongational Viscosity

The rheology die we developed can only be used to measure shear viscosity. For comparison, we employed a second rheology die for extruders (property of the Institute of Polymer Extrusion and Compounding, Johannes Kepler University Linz, Austria), which was developed by Luger et al. [22] and has two slit sections and a contraction section in between (the theory on elongational viscosity briefly presented below comes from this publication). The slit sections allow measurement of shear viscosity, and a WR correction as given by Equations (5)–(8) is required. In the contraction section, both shear and elongation flows occur. Pressures are measured in the two slit sections. The dimensions of the viscosity die are provided in Table 2.

In contrast to shear viscosity, elongational viscosity is a function of the average elongation rate, $\dot{\varepsilon }$, in the contraction section. It can be calculated by

(13) $$\dot{\varepsilon }=\frac{\dot{V}}{l_{c}}\left(\frac{1}{w_{2}h_{2}}-\frac{1}{w_{1}h_{1}}\right)\;$$

where $\dot{V}$ represents the volumetric flow rate.

The measured pressure gradient over the contraction section, $\Delta p_{meas.}$, is the sum of pressure losses due to viscous shear flow, $\Delta p_{shear}$, and the entrance pressure drop, $\Delta p_{E}$. Elongational viscosity can be calculated by

(14) $$\eta_{elong.}=\frac{\Delta p_{E}}{\dot{\varepsilon }}=\frac{\Delta p_{meas.}-\Delta p_{shear}}{\dot{\varepsilon }}\;$$

The contribution from viscous shear flow can be obtained from

(15) $$\frac{\Delta p_{shear}}{z}={\left(\frac{2^{m+1}\left(m+2\right)\dot{V}}{w\left(z\right)h{\left(z\right)}^{m+2}\phi }\right)}^{\frac{1}{m}}$$

where *z* denotes the longitudinal coordinate in the flow direction (0 ≤ z ≤ $l_{c}$), w(z) and h(z) are width and height of the contraction section, ϕ is the fluidity, and m is the flow index for power-law fluids.

### 3.4. Ultrasound Measurements of Volumetric Flow Rate

In viscosity measurements using capillary rheometers, the volumetric flow rate in the die is usually calculated from the displacement of a plunger or (in the case of measurements on an injection molding machine) the screw. Therefore, as mentioned earlier, potential sources of error are a malfunctioning backflow barrier and increased compressibility of gas-containing polymer melts. To accurately measure volumetric flow rates directly in the channel of our die, ultrasound sensors are used. These measurements are facilitated by the rectangular die and channel geometry.

Prior to the ultrasound measurements, the reproducibility of volumetric flow rates realized by the injection-molding machine was verified by comparing actual shot weights and oscilloscope recordings of the machine. On this basis, the capabilities of ultrasound measurement technology were assessed.

For the gravimetric measurements, a shot volume of 100 cm^3^ and a constant backpressure of 140 bar were set. The volumetric flow rate, ${\dot{V}}_{set}$, was set to 5, 10, and 20 cm^3^ s^−1^, and agreement with the actual flow rates was assessed by measuring the ejection times. 20, 10, and 5 s were expected, respectively. Additionally, given the density of the material (from prior pvT measurements), the expected theoretical shot weights were compared to the actual weights of ejected material.

Two ultrasound sensors were attached along the flow channel on the outside of the die, as indicated in Figure 1. With the die in this configuration, the distance between the sensors was 6 cm, which was too large for a cross-correlation approach using the neat polymer melt. We therefore used a small number of tracer particles made of heat-resistant silicone. Five to ten markers with diameters below 0.5 mm were added via the hopper and transported along with the polymer being processed. The sampling rate of both sensors was set to 250 s^−1^ to provide sufficient resolution.

## 4. Materials and Equipment

Rheological measurements in injection molding using our slit die were carried out on an Engel EM 310/100 T injection-molding machine (Engel Austria GmbH, Schwertberg, Austria). For reference measurements of the neat polymer, an HPCR Rheograph 25 (Göttfert Werkstoff-Prüfmaschinen GmbH, Buchen, Germany) was employed, which is quite similar to an injection molding plasticizing unit in terms of the main measurement setup (axially moving piston and temperature-controlled barrel). Rheometric tests in extrusion were performed on a HAAKE PolyLab OS single-screw extruder (Thermo Fisher Scientific Inc., Waltham, MA, USA).

The materials for injection molding and extrusion are summarized in Table 3. Two PP homopolymers (PPH-1 and PPH-2) with melt flow rates of 12 g/10 min and 8 g/10 min (230 °C/2.16 kg), respectively, were used as received.

Two endothermic CBAs were used in this study: CBA-1 has 40% active components and a decomposition temperature of 160 °C, while CBA-2 (the masterbatch) consists of 50% active components and requires a minimum processing temperature of 220 °C. Both CBAs yield CO_2_, but the quantities are not specified by the suppliers.

## 5. Results and Discussion

### 5.1. Thermal Characterization and Pressure Calibration

**Figure 4:**
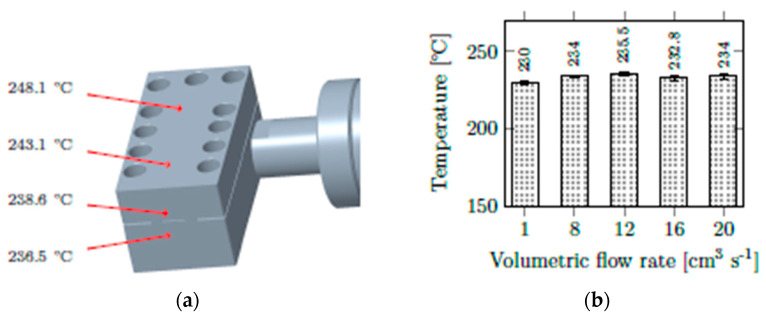
(**a**) Surface temperatures of the rheology die and (**b**) temperatures of the ejected material at various flow rates.

Before the experiments with CBAs, our rheology die for injection molding had to pass thermal characterization tests. The die was heated via a heater band controlled by the injection-molding machine. A temperature of 230 °C was set. Several consecutive shots (shot volume: 50 cm^3^) of PPH-2 were ejected through the mounted die at various volumetric flow rates. At each flow rate, surface temperature at several positions (as indicated in Figure 4a) and the temperature of the ejected material were measured for every 10th shot. Surface temperature was measured using a crossband sensor, while the melt temperature was determined by a rod-type temperature sensor measuring the ejected material collected in an insulated polytetrafluoroethylene (PTFE) bin. The results of this temperature characterization are summarized in Figure 4. As can be seen, temperature regulation worked properly. Surface temperatures were slightly elevated, but the exiting melt had the desired temperature.

**Figure 5:**
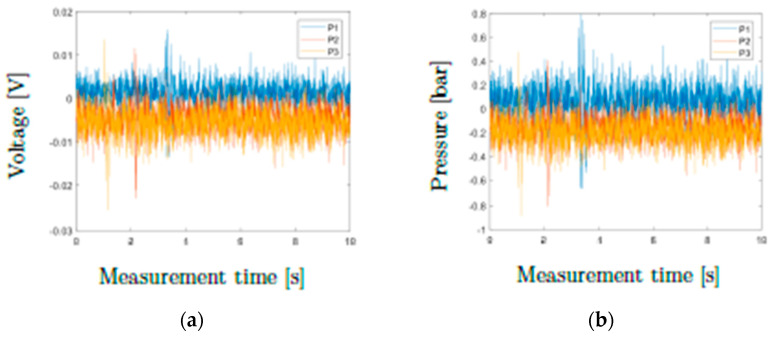
(**a**) Voltage and (**b**) pressure signals of the pressure transducers in a zeropressure state (calibration).

Subsequently, the three pressure transducers were calibrated. At position P1 (top) a 500-bar pressure sensor and at positions P2 and P3 (middle, bottom) 350-bar pressure sensors were mounted. For calibration, the screw channel was emptied, and a zero-pressure state was realized in the die. All sensors were calibrated such that the recorded pressures were in the range between −1 and +1 bar. Figure 5 shows the recorded voltages and the corresponding pressures over a measurement time of 10 s. This calibration was performed before each series of tests. After successful thermal characterization and pressure calibration, our rheology die was ready for use in viscosity measurements.

### 5.2. Rheology Measurements with CBA—Shear Viscosity

For assessing the influence of gas concentration on the viscosity of polymer melts, the two CBAs from Table 3 were used in combination with PPH-1. Measurements were carried out at various concentrations and pressure gradients (i.e., pressure drops between P1 and P2; P1and P3; and P2 and P3). The temperature was again set to 230 °C.

#### 5.2.1. Choice of Suitable Correction Approach for Rheology Die

Before starting the test series, we had to choose an appropriate correction approach from those described in Section 3.2. For this purpose, we applied the three approaches to data from viscosity measurements of PPH-1 without gas using pressures P2 and P3. To improve the capabilities of these approaches (i.e., to reduce inaccuracies when measurement points are too wide apart), the measurement data were approximated with a Bird-Carreau-Yasuda viscosity model according to Equation (16), which ignores temperature dependency [23]. The four fitting parameters are the viscosity at zero shear rate, $\eta_{0}$, the relaxation time, λ, and two dimensionless factors, a and n. Once a proper fit has been found, gradients of apparent shear rate—or viscosity for approaches 1 and 3—can be calculated.

(16) $$\eta =\frac{\eta_{0}}{{\left[1+{\left(\lambda {\dot{\gamma }}_{ap}\right)}^{a}\right]}^{\frac{1-n}{a}}}\;$$

**Figure 6:**
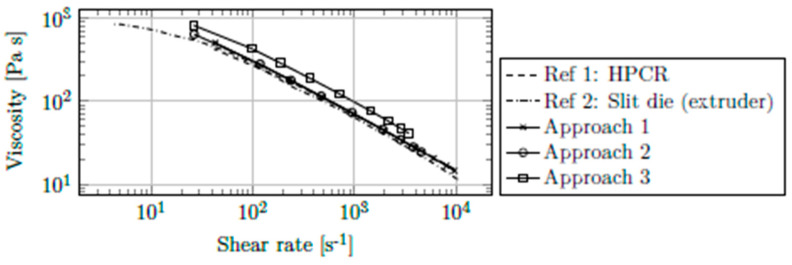
Viscosity of PPH-1 at 230 °C corrected by WR correction (Approach 1), Schümmer approximation (Approach 2), and a variant of WR correction (Approach 3), and reference measurements (ref. [1]: HPCR, ref. [2]: extrusion die).

Comparative measurements were necessary to assess each correction approach. Figure 6 includes reference measurements of the neat polymer using HPCR ([1]) and the extrusion die ([2]; also capable of measuring elongational viscosity) and the results from the injection molding die corrected by the three candidate approaches. Both reference measurements were corrected with the WR approach.

Clearly, the WR correction (approach 1) and the Schümmer approximation (approach 2) provided the most accurate corrections of raw data. As can be seen, WR correction shifts the apparent viscosity curve to higher shear rates and therefore lower viscosities. The Schümmer approximation only assigns corrected shear rates to apparent viscosities and thus shifts the apparent curve horizontally to the left. Using a variant of WR correction (approach 3) positions the corrected viscosity curve approximately 30% too high. The standard WR correction (approach 1) was therefore used for further data evaluation.

#### 5.2.2. Measurement Results

**Figure 7:**
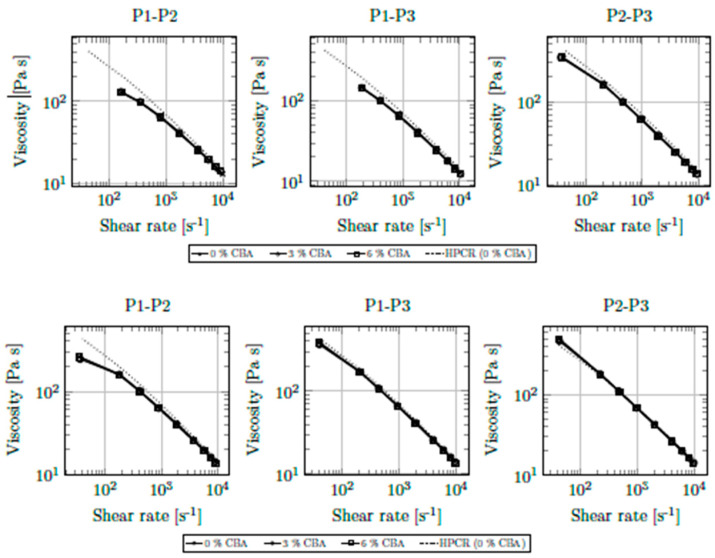
Shear viscosity of PPH-1 at 230 °C and CBA contents of up to 6% (upper: CBA-1, lower: CBA-2). Dotted lines: HPCR reference measurements.

Various combinations of pressure transducers can be used to calculate viscosities. Figure 7 shows corrected results from viscosity measurements of PPH-1 with CBA contents (either CBA-1 or CBA-2) of up to 6%. For reference, the results of HPCR without gas at 230 °C are included.

Again, good agreement with HPCR measurements was achieved for all combinations of pressure transducers. Only at the lowest shear rates (and especially when using pressure transducers P1 and P2) did deviations from the reference curve occur. The pressure transducer mounted at position P1 was identified as the source of this error. Since the largest pressure occurs at the beginning of the flow channel, this transducer has the widest pressure range of 0–500 bar (compared to 0–350 bar at P2 and P3). At the lowest volumetric flow rate of 0.1 cm^3^ s^−1^ (i.e., at a corrected shear rate of 40 s^−1^), pressures below 20 bar act at position P1. With an accuracy of ±0.5% of the full-scale output of 10.5 V, this may cause scattering of up to ±25% around the true pressure. Obviously, the pressure sensor with the widest range has the largest error. This disqualifies our rheology die from use with extremely low shear rates. However, especially in the injection stage, such shear rates are of negligible importance.

Contrary to our expectations, neither CBA had an appreciable influence on the rheological properties of the polymer. Irrespective of the CBA used, even the highest content of 6% did not improve the flow behavior of the material. The vast majority of scientific publications that describe the influence of gases on the viscosity of polymer melts have investigated physical foaming. In addition, a huge number of different (exothermic and endothermic) CBAs are commercially available. Although Qin et al. [13] reported considerable viscosity reductions using CBAs, it is very likely that the compositions of their blowing agents (active constituents, carrier polymer, gas yield, etc.) were different and not comparable to those in our study. Our results indicate that the gas concentration yielded from CBA decomposition is insufficient to influence the flow behavior of the polymer melt.

We subsequently assessed in greater mathematical detail the (presumably small) influence of CBAs on the flowability of the polymer melt. Application of the Bird-Carreau-Yasuda viscosity model enabled precise description of corrected viscosity measurements. For all fits, correlation coefficients greater than 0.9999 were achieved. The parameters of the viscosity model can be found in Table A1 in the Appendix A. With these curves, it became possible to visualize the viscosity reduction associated with each CBA concentration. Shear rates under 10^2^ s^−1^ were ignored due to the inaccuracies discussed above. Table 4 summarizes the results.

With only one exception, a viscosity reduction resulting from CBA addition could be shown. Unsurprisingly, the most beneficial influence was seen for the largest CBA concentration of 6%. The positive influence, however, seemed to level off, as the plasticizing effect was approximately three times larger between 0% and 3% CBA than between 0% and 6%. Interestingly, comparison of the two blowing agents showed a considerably greater plasticizing effect of CBA-2 despite the larger proportion of active components in CBA-1. No trend regarding individual shear rates could be observed, which is why Table 4 includes only average values.

### 5.3. Rheology Measurements with CBA—Elongational Viscosity

Since only small improvements in shear viscosity were observed (using our rheology die for injection molding processes), elongational viscosity was investigated next. Elongational flows are inevitable in foam injection molding: During cell growth, the material is elongated along the cell walls, which can (in extreme cases) cause rupture of the cell wall or a fibril structure in the core of a foamed component [24]. It has been shown that the counter-pressure that acts on the melt front has a significant influence on both expansion ratio and cell population density [25]. When nuclei have already started to form in the mold-filling stage, there is a chance that the elongational flow will influence the flow behavior of gas-containing polymer melts.

#### Measurement Results

For measurements of elongational viscosity, a special die was mounted on an extruder. To obtain a stable flow, a melt pump with a throughput of 2400 mm^3^ r^−1^ was installed. The pump was operated at rotational speeds between 1 and 25 rpm, which corresponds to elongational rates between 3 and 75 s^−1^. Only CBA-1 was used as a blowing agent in these trials.

**Figure 8:**
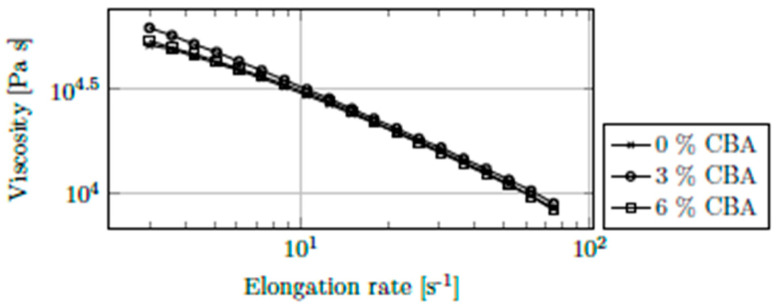
Elongational viscosity of PPH-1 at 230 °C and CBA contents of up to 6% (CBA-1).

As illustrated in Figure 8, just as with shear viscosity, elongational viscosity also decreased with increasing elongation rate, which has also been shown in other studies [26,27]. Again, no influence of the CBA content on viscosity was observed. Application of mathematical viscosity models, as described above, was not useful, as no trend regarding the influence of the CBA content was identified. 3% CBA led to the highest viscosity, while a comparison between 0% and 6% suggests that high CBA contents have a beneficial influence only at low elongation rates. These inconsistencies lead to the assumption that the influence of CBAs on elongational viscosity is negligible and that the variation in elongational viscosities is due to measurement errors or process instabilities.

As noted by Rauwendaal [28] (p. 205), elongational viscosities are considerably higher than shear viscosities. One way of comparing elongational and shear viscosity is to use the Trouton ratio (i.e., the ratio of elongational viscosity to shear viscosity). As stated in the literature, the Trouton ratio is always equal to 3 for Newtonian fluids [29,30]. However, it was shown by Binding et al. [31] and Bach et al. [32] that the ratios for polymeric materials are larger by a factor of 10–100.

**Figure 9:**
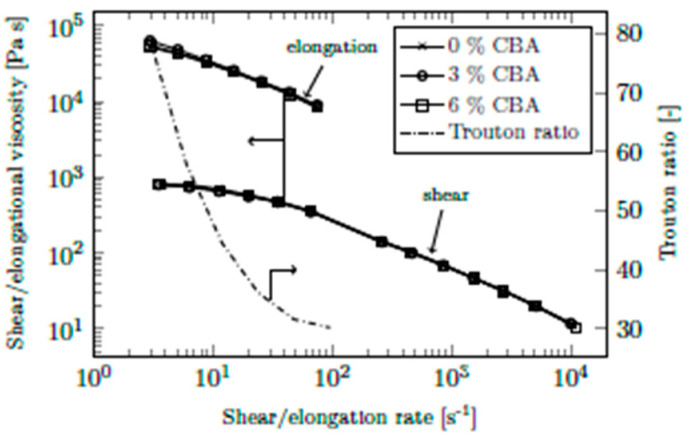
Comparison of elongational and shear viscosity of PPH-1 at 230 °C, including the Trouton ratio (CBA-1). The arrows indicate the corresponding *y*-axis.

As plotted in Figure 9, we obtained Trouton ratios between 80 and 30 at shear/elongational rates between 3 s^−1^ and 10^2^ s^−1^. This order of magnitude is in good agreement with that reported by Binding et al. [31]. The comparison of the two flow types and the good accordance between literature and measured values confirm the validity of our rheology die for injection molding. No noticeable influence of CBAs on either type of viscosity was identified.

### 5.4. Ultrasound Measurements of Volumetric Flow Rate

**Figure 10:**
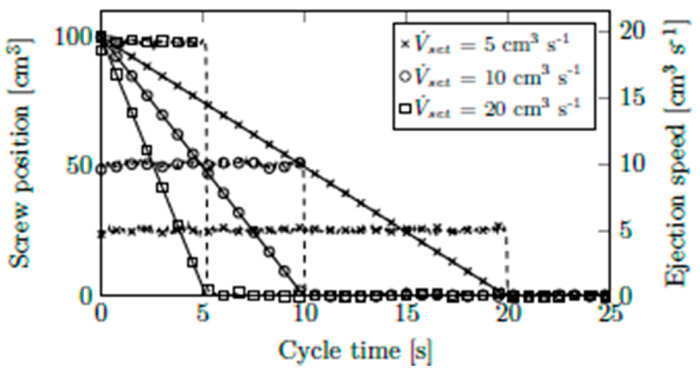
Oscilloscope recordings of screw position (solid lines) and speed (dashed lines) at various volumetric flow rates.

We developed our rheology die with the aim of enabling the innovative measurement of volumetric flow rate via ultrasound sensors. Neat PPH-1 without addition of CBAs was used in these experiments. First, reproducibility and theoretical output were analyzed by investigating oscilloscope recordings. For each of the three volumetric flow rates, ${\dot{V}}_{set}$, (i.e., set to 5, 10, and 20 cm^3^ s^−1^), Figure 10 plots screw position and ejection speed. Each setting was run five times. The curves were virtually congruent, which is why only one curve is shown per setting. As can be seen, both screw position and speed were controlled accurately. The expected ejection times (calculated from plasticized shot volume and set volumetric flow rate) corresponded perfectly with the actual ones.

This machine performance is necessary for precise control of the volumetric flow rate, but does not prove it. For instance, an improperly shutting backflow barrier could still cause backflow. All shots were therefore weighed, and their calculated volumes were compared to the shot volumes from the machine. The material density at 140 bar and a processing temperature of 230 °C was found to be 1.37 g cm^−3^. Average shot weights, $\bar{w}$, calculated shot volumes, $\bar{V}$, the corresponding standard deviations, $\sigma_{w,V}$, average ejection time and its standard deviation, $\bar{t}$ and $\sigma_{t}$, and the calculated average volumetric flow rate and its standard deviation, $\bar{\dot{V}}$ and $\sigma_{\bar{\dot{V}}}$, are summarized in Table 5. This analysis clearly shows that no unwanted leakage or other sources of error negatively affected the accuracy of volumetric flow rates.

**Figure 11:**
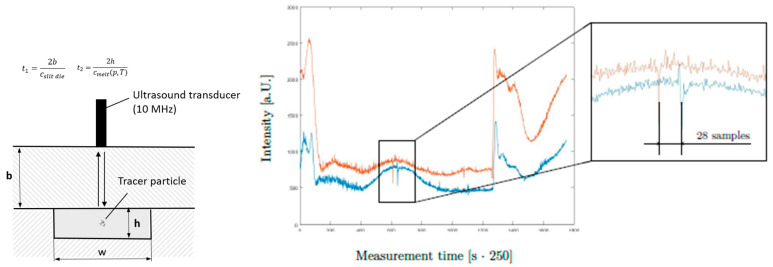
Ultrasound-based determination of volumetric flow rate at a sampling rate of 250 s^−1^. The parameter c describes the speed of sound.

This verification was used as a basis for subsequent ultrasound measurements. The sample rate of 250 s^−1^ in combination with the time that it takes the tracer particles to flow from the first to the second sensor allowed volumetric flow rates to be calculated. When a particle passes a sensor, it absorbs and scatters the ultrasound signal for a short period of time. The example in Figure 11 plots the intensity of the ultrasound signal over measurement time. The upper and lower curves show measurements from the first and second sensors, respectively. The moment a tracer particle passed one of the sensors is visible as a sharp downward peak.

In this example, a volumetric flow rate of 10 cm^3^ s^−1^ was set. As can be seen, a tracer particle passes the two sensors at different times. The distance between the two peaks can be translated into a time period and, ultimately, into a volumetric flow rate. At a sample rate of 250 s^−1^, 28 samples that are recorded while the particle flows from one sensor to the other represent a time period of 0.112 s. Combining this value with the geometry of the flow channel yields a volumetric flow rate of 10.7 cm^3^ s^−1^. This result accords well with both our expectations and the calculations in Table 5. Provided that the sampling rate can be increased to improve resolution, ultrasound measurement has real potential for direct determination of volumetric flow rates.

## 6. Conclusions

We have presented a novel measurement die for near-process investigation of viscosity. Our die can be mounted directly on an injection molding machine and helps to overcome problems related to investigating gas-containing polymer melts (i.e., maintenance of sufficient pressure). Reference measurements using an HPCR and neat PPH-1 confirmed the accuracy of our measurement device. Interestingly, use of two different endothermic CBAs had minimal influence on shear viscosity. Only 2.07% and 3.10% reductions in viscosity were achieved with CBA-1 and CBA-2, respectively. We assume that the gas yield from the CBAs is was insufficient to have a significant impact on viscosity.

To confirm that the gases have negligible influence on the flow behavior, measurements of elongational viscosity in an extrusion process were conducted. Investigating the same polymer with one of the blowing agents (CBA-1) confirmed our results from shear viscosity. No reduction in viscosity, even with a blowing agent content of 6%, was observed, making it necessary to perform similar investigations in physical foaming with both N_2_ and CO_2_ and with higher gas contents in general. To this end, the die will be adapted for mounting on relevant machines.

A drawback of the current configuration of the die is its inability to accurately monitor very low shear rates, which would require installation of suitable pressure transducers, which are also suitable for non-static applications However, with the pressure sensor used (0…500 bar), an accuracy of +/−1.25 bar is already very good. A better alternative could be the use of ultrasonic sensors for pressure measurement, but this involves considerable effort in material characterization, in order to create suitable calibration curves.

Our die allows the volumetric flow rate in the flow channel to be monitored, and we have shown that it can be used with ultrasound sensors. In its current configuration, it requires tracer particles. As part of future work, a denser sensor arrangement will be realized, which should remove the need for tracer particles. This novel use of ultrasound holds great promise for viscosity measurement.

## Figures and Tables

**Table 1:** Dimensions of slit.

Parameter	Dimension
Width, w	20 mm
Height, h	1 mm
Total die length	120 mm
Distance between pressure transducers	30 mm
Distance from inlet to first transducer	30 mm
Distance from last transducer to outlet	30 mm

**Table 2:** Dimensions of the rheology die for extrusion.

Section	Parameter	Dimension
Slit Section 1	Height, $h_{1}$	2 mm
	Width, $w_{1}$	20 mm
	Length, $l_{1}$	65 mm
Slit Section 2	Height, $h_{2}$	0.5 mm
	Width, $w_{2}$	5 mm
	Length, $l_{2}$	5 mm
Contraction section	Length, $l_{c}$	5 mm

**Table 3:** Summary of the materials used in this study.

	Injection Molding	Extrusion	Supplier
**Polymer**	PPH-1	PPH-1	Borealis Polyolefine GmbH, Linz, Austria
	PPH-2 ^1^		
**CBA**	CBA-1	CBA-1	Trexel, Inc., Wilmington, MA, USA
	CBA-2		Clariant AG, Muttenz, Switzerland

^1^ For thermal characterization and temperature study only.

**Table 4:** Reduction in shear viscosity due to CBAs at shear rates between 10^2^ and 10^4^ s^−1^ (values in %).

	CBA-1				CBA-2			
Content Range	P1-P2	P2-P3	P1-P3	Grand Average	P1-P2	P2-P3	P1-P3	Grand Average
**0–3**	0.34	−2.97	−1.92	**−1.52**	−2.60	−2.40	−2.39	**−2.47**
**3–6**	−0.88	−0.05	−0.76	**−0.56**	−0.78	−0.20	−0.98	**−0.65**
**0–6**	−0.52	−3.04	−2.66	**−2.07**	−3.36	−2.60	−3.35	**−3.10**

**Table 5:** Weights and calculated volumes of ejected material and ejection time per set volumetric flow rate.

${\dot{V}}_{set}$	$\bar{w}$	$\bar{V}$	$\sigma_{w,V}$	$\bar{t}$	$\sigma_{t}$	$\bar{\dot{V}}$	$\sigma_{\bar{\dot{V}}}$
[cm^3^ s^−1^]	[g]	[cm^3^]	[%]	[s]	[s]	[cm^3^ s^−1^]	[cm^3^ s^−1^]
5	74.13	101.56	0.28	19.89	0.02	5.11	0.02
10	73.90	101.24	0.36	9.95	0.00	10.18	0.04
20	73.97	101.33	0.16	5.15	0.00	19.68	0.03
Average	74.00	101.38	0.26				

## Data Availability

The data presented in this study are available on request from the corresponding author.

## Appendix A

**Table A1:** Parameters of the Bird-Carreau-Yasuda viscosity model for PPH-1 with various CBA contents at 230 °C.

			CBA-1			CBA-2		
			P1-P2	P2-P3	P1-P3	P1-P2	P2-P3	P1-P3
0%	$\eta_{0}$	[Pas]	209.89	380.56	998.85	282.03	1235.14	741.10
	λ	[s]	0.0040	0.0174	0.0057	0.0114	0.0742	0.0368
	a	[−]	0.8248	2.1379	0.3398	1.6397	1.2022	0.9208
	n	[−]	0.2539	0.3556	0.0750	0.3674	0.3305	0.3312
3%	$\eta_{0}$	[Pas]	168.33	440.35	313.72	256.21	944.15	563.97
	λ	[s]	0.0049	0.0201	0.0089	0.0101	0.0470	0.0247
	a	[−]	1.4388	1.3521	0.8621	1.8405	1.1502	1.0644
	n	[−]	0.3525	0.3378	0.2902	0.3650	0.3185	0.3267
6%	$\eta_{0}$	[Pas]	171.12	427.71	331.75	286.25	1237.81	784.27
	λ	[s]	0.0053	0.0207	0.0100	0.0118	0.0723	0.0362
	a	[−]	1.3787	1.6147	0.8235	1.5013	1.0608	0.8040
	n	[−]	0.3595	0.3469	0.2953	0.3597	0.3220	0.3117
